# An efficient LSPR method to quantitatively detect dimethoate: Development, characterization and evaluation

**DOI:** 10.1371/journal.pone.0239632

**Published:** 2020-09-24

**Authors:** Dongxian Li, Yanyan Zhang, Qingqian Guo, Xiaoquan Sun, Hao Zhang, Shun Wang, Zephania Birech, Jiandong Hu

**Affiliations:** 1 Department of Electrical Engineering, Henan Agricultural University, Zhengzhou, China; 2 Henan International Joint Laboratory of Laser Technology in Agriculture Sciences, Zhengzhou, China; 3 Henan Institute of Metrology, Zhengzhou, China; 4 College of Science, Henan Agricultural University, Zhengzhou, China; 5 Department of Physics, University of Nairobi, Nairobi, Kenya; 6 State Key Laboratory of Wheat and Maize Crop Science, Zhengzhou, China; University of Southern Denmark, DENMARK

## Abstract

In recent years, there has been growing concern among consumers about pesticide contamination in fruits. Therefore, rapid, reliable, and consistent detection methods for OPPs, especially dimethoate, are crucially needed. The existing quantitative methods for detecting dimethoate are not suitable for rapid measuring system such as the dimethoate samples from two channels. Hence this paper examines the utilization of a dual-channel system for utilize the absorption variations of the Localized Surface Plasmon Resonance (LSPR) bands of gold nanoparticles (AuNPs) were investigate for detection of dimethoate. Under optimized conditions, the relationship between concentrations of dimethoate and absorbance ratios (A(520)/A(640)) was linearly found in the concentration range of 10–100 nM. Result from the experiment shows that both channels exhibit a linear correlation coefficient as high as 0.97 and a limit of detection (LOD) as low as 5.5 nM. This LSPR detection system was characterized by testing the dimethoate in apple samples and the recovery rates were found to be in the range of 85.90% to 107.37%. The proposed dual-channel LSPR system for detecting dimethoate creating a new approach for detecting organophosphate insecticide in agricultural fields. It could lay the foundation for designing a high-throughput analysis of the insecticides using a wavelength division multiplexing switch (WDMS).

## Introduction

The applications of insecticides to suppress mole crickets are the most effective approaches to improve the production of field crop for a long time. However, prolonged and uncontrolled use of these insecticides results in their residues getting transported to areas away from where they were applied thus posing environmental, animal, and human health risks. Some amount of the applied pesticides can remain in the fields for a considerable length of time [1–3]. Pesticides are widely used in agriculture, and therefore have become a public concern. Reportedly, pesticide has been considered to be a worldwide pollution of agricultural due to its chemical contamination, especially in developing regions. China is the world’s largest producer and consumer of chemical pesticides [4]. According to statistics, the amount of chemical pesticides used per unit of cultivated land in China is about 2.5 to 5 times the world average, and the amount of pesticides actually used by farmers was estimated to be 2.8, 2.3 and 1.2 times the optimal level in cotton, rice and corn production respectively [5]. A wide variety of insecticide dimethoate 40% or 50% in emulsifiable concentration are available to the market. Because toxicity and the bioaccumulation effect, dimethoate is moderately toxic by ingestion, inhalation and dermal absorption. People with respiratory ailments, recent exposure to cholinesterase inhibitors, impaired cholinesterase production, or with liver malfunction may be at increased risk from exposure to dimethoate [6–8]. Dimethoate is one of the commonly used pesticides in the field of avocado, cereal, citrus, cotton, mango, peanut and pulse crops [9, 10]. Dimethoate (C_5_H_12_NO_3_PS_2_) is a colorless crystalline solid in its pure form and is soluble in chloroform, methylene chloride, benzene, toluene, alcohols, esters, ketones and partly in water [11]. It is therefore not surprising that rapid increase in the development of highly efficient methods of dimethoate characterization, quantification, and detection techniques has been seen. Traditional detection methods of dimethoate residues such as colorimetric method [12], immunoassay [13], chromatography [14], chromatography-mass spectrometry [15] and high-performance liquid chromatography coupled-tandem mass spectrometry (LC-MS/MS) [16, 17] have some limitations. These limitations include complicated and time-consuming sample pre-treatment processes, but also involve expensive instruments only operated by trained personnel. Obviously, those expensive instruments cannot be effectively implemented to establish a quick determination method of analytes in the field [18, 19].

Research into development of alternative devices or system has been on-going. Fluorescence spectroscopic method was reported by Nian et al. provided a sensitive solution for detecting organophosphorus pesticides where it inhibits Acetylcholinesterase (AChE) activity and catalyzes the carbamide reaction with copper [20]. AChE is a type of enzyme with significant role in the nervous system. Liang et al. carried out synthesis of three haptens for the class-specific immunoassay of O-dimethyl organophosphorus pesticides [21]. Immunoassay has high sensitivity and specificity, but the preparation of antibodies is complicated and expensive technique [22]. Although LC-MS/MS has high accuracy and sensitivity, the instruments are expensive and bulky. Therefore, a rapid detection method with high specificity and sensitivity for detecting dimethoate on-sites is urgently needed. Localized Surface Plasmon Resonance (LSPR) [23] is a phenomenon that increase the utilization of biosensing field due to its potential application in trace molecule and label free detection. Noble metal nanomaterials with strong LSPR characteristics have become an effective and rapid tool for the detection of pesticide residues with high sensitivity, high selectivity and low interference [24]. Compared with Surface Plasmon Resonance (SPR) for the detection of pesticide residues, Localized Surface Plasmon Resonance (LSPR) of metallic nanoparticles is advance technique for biosensing due to its stable LSPR signals from the easily fabricated metal nanoparticles. In order to improve the repeatability and sensitivity of active substrates for the LSPR detection, researchers have used different nanostructured metal particles [25, 26]. Du et al. prepared a polymer film in which silver nanoparticles (AgNPs) was synthesized to detect dimethoate [27]. The experimental results showed that the LSPR peak responses were proportional to the concentrations of dimethoate in the range of 1.0–1000 ng/mL and even in the range of 1.0–50 ng/mL with a limit of detection (LOD) of 0.5 ng/mL. Bai et al. put aptamers to detect organophosphorus pesticides indirectly due to these stable compounds coated with gold nanoparticles (AuNPs) in salt solution [28]. By adding dimethoate, the aptamers were separated from AuNPs surface and the AuNPs were aggregated. Correspondingly, the color of the solution was changed from red to purple blue. The recovery rate of 72%-135% was achieved. However, the number of works devoted to the rapid simultaneous multi-element determination of dimethoate with low cost is limited.

Here, we report a novel dual-channel LSPR technology for the quantification of the dimethoate based on an aggregation of AuNPs. Normally, the surface of the synthesized AuNPs carries negative charge and the color of the AuNPs solution is wine-red. The AuNPs were aggregated in the presence of NaOH and then the color was turned into grey-blue due to the electrostatic repulsion between the negative charges on the surface of AuNPs induced by the high concentration of NaOH. Dimethoate can be easily hydrolyzed under strong alkaline conditions and the hydrolyzed product carries negatively charges. Accordingly, the AuNPs were well-dispersed due to the hydrolyzed product with strong negative charges after adding NaOH to the mixed solution of dimethoate and AuNPs. This method was used to quantify dimethoate with good in-laboratory reproducibility, and the matrix effect was assessed from the dimethoate concentrations spiked in apple juice. Additionally, the identity of dimethoate was confirmed by electrochemical workstation and fluorescent hydrogel and Surface Enhanced Raman Scattering (SERS). These studies strongly support our envisages of high-throughput LSPR analysis of the insecticides using a wavelength division multiplexing switch (WDMS) in the future.

## Materials and methods

### Reagents

Dimethoate, hexachloro-cyclohexane soprocide, fenvalerate and ethoprophos were purchased from Aladdin Reagent Co. Ltd. (Shanghai, China). Tetrachloroauric (III) acid tetrahydrate (HAuCl4·4H2O) and trisodium citrate were acquired from Sigma-Aldrich (USA). NaOH and methanol were supplied by Sinopharm Chemical Reagent Co. Ltd. (Shanghai, China). Chemicals for analytical reagents grade were used without further purification. Milli-Q water was used in all sample solution throughout.

### Fabrication of dual-channel LSPR system

#### Experimental realization

**Fig 1:**
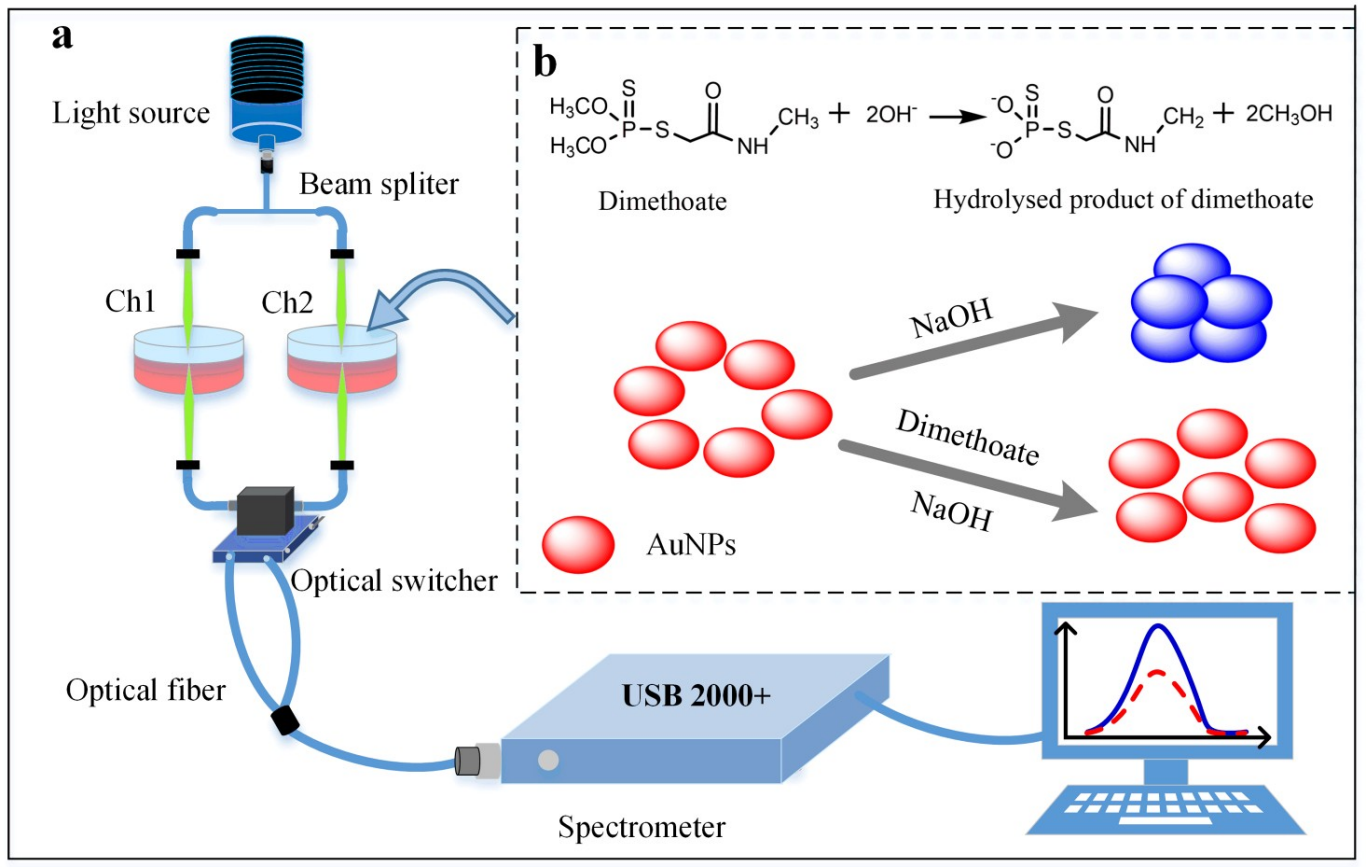
Schematic illustrating the detection principle of dimethoate using the dual-channel LSPR system via optical fibers. The dual channel LSPR device is shown in (a), detection principle inset (b).

The schematic diagram of the dual-channel LSPR system is shown in Fig 1a. This dual-channel LSPR system consists of a broadband light source (tungsten halogen lamp) with a spectral range of 200 to 1700 nm, two premium bifurcated optical fibers with SMA905 connectors, an Optical Fiber Dual Switch (OFDS) and a miniature spectrometer of USB 2000+ with a linear Charge Coupled Device (CCD) array (Optic Oceans). By manually adjusting OFDS, the spectral data from both channels were displayed graphically on the computer. The measured sample is poured into the cuvette embedded in the holder, the light beam from the tungsten halogen lamp is well-distributed by the beam splitter to travel through two different pathways named as channel 1 (Ch1) and channel 2 (Ch2) via optical fibers. The spectral signals coming out from the cuvette are time-division transferred by the optical switch and dispersed by the USB 2000+ spectrometer then the spectral signal of the measured sample is displayed on the computer.

The detection principle of dimethoate is illustrated in the inset Fig 1b. The surface of the synthesized AuNPs carries negative charge and the color of the AuNPs solution is wine-red. The AuNPs are well dispersed due to the electrostatic repulsion between citrate roots. After a known concentration of NaOH solution is added into the AuNPs solution, the AuNPs are aggregated and then the color turns into grey-blue due to the screening of the negative charges on the surface of gold nanoparticles by the high concentration of NaOH. Obviously, a high concentration of NaOH can make the AuNPs aggregate quickly, but a low concentration of NaOH was not obvious and even could not be induced aggregation of the AuNPs. However, dimethoate is easily hydrolyzed under the strong alkali condition, resulting in the product carrying negative charges. Consequently, the phenomenon of anti-aggregation of AuNPs occurred due to the electric repulsion force between the negative charges of hydrolysate of dimethoate and the negative charges of the citrate radical. Therefore, by adding dimethoate with different concentrations into the solution formed by AuNPs and NaOH mixture, anti-aggregation of AuNPs is achieved at different degrees and the color of the mixture changes from blue to red.

#### Synthesis of the citrate-stabilized AuNPs

The citrate-stabilized AuNPs with an average diameter of 13.5 nm were synthesized by sodium citrate reduction [29, 30]. Briefly, 25 mL of HAuCl4 aqueous solution (1 mM) was brought to boil for 2 min in a round-bottom flask. Then, quickly 2.5 mL of a 1% solution of sodium citrate (C6H5Na3O7·2H2O) was added into this solution and let it react for 5 min. After that, it was kept boiling for 10 min until the color of the solution turned into wine-red. The morphology and particle size of prepared AuNPs were characterized by a JEM-2100 high-resolution transmission electron microscopy (TEM). The ultraviolet-visible (UV-Vis) absorption spectrum of AuNPs was recorded by using a UV-Vis spectrophotometer (Nanjing Philes Instruments Co., Ltd., China). The citrate stabilized AuNPs with the concentration of 9.5 nM was obtained and stored at 4°C for further use.

## Results and discussion

### Preparation of measured solutions

For the rapid detection of dimethoate, 80 μL of dimethoate aqueous solutions at different concentrations of 10 nM, 30 nM, 50 nM, 80 nM and 100 nM were placed into a centrifuge tubes containing 535 μL of Milli-Q water, respectively. 35 μL of NaOH (1.5 M) was added into these tubes, the AuNPs solution were also added to the mixture, then after 10 min reaction, 330 μL of the mixture was transferred into a cuvette embedded inside the holder. The dimethoate detection was performed by the dual-channel LSPR system via optical fibers. The developed dual-channel LSPR system was applied in rapid detection of dimethoate in apples. The apples were cut into pieces and ground to form the apple juice. The juice was then centrifuged at a speed of 12000r/min for 10 min. The supernatant was sieved with a 0.22 μm filter. Afterwards, the ensured samples for performing these detection experiments were spiked with known concentration of dimethoate solutions.

### Characterization of AuNPs

**Fig 2:**
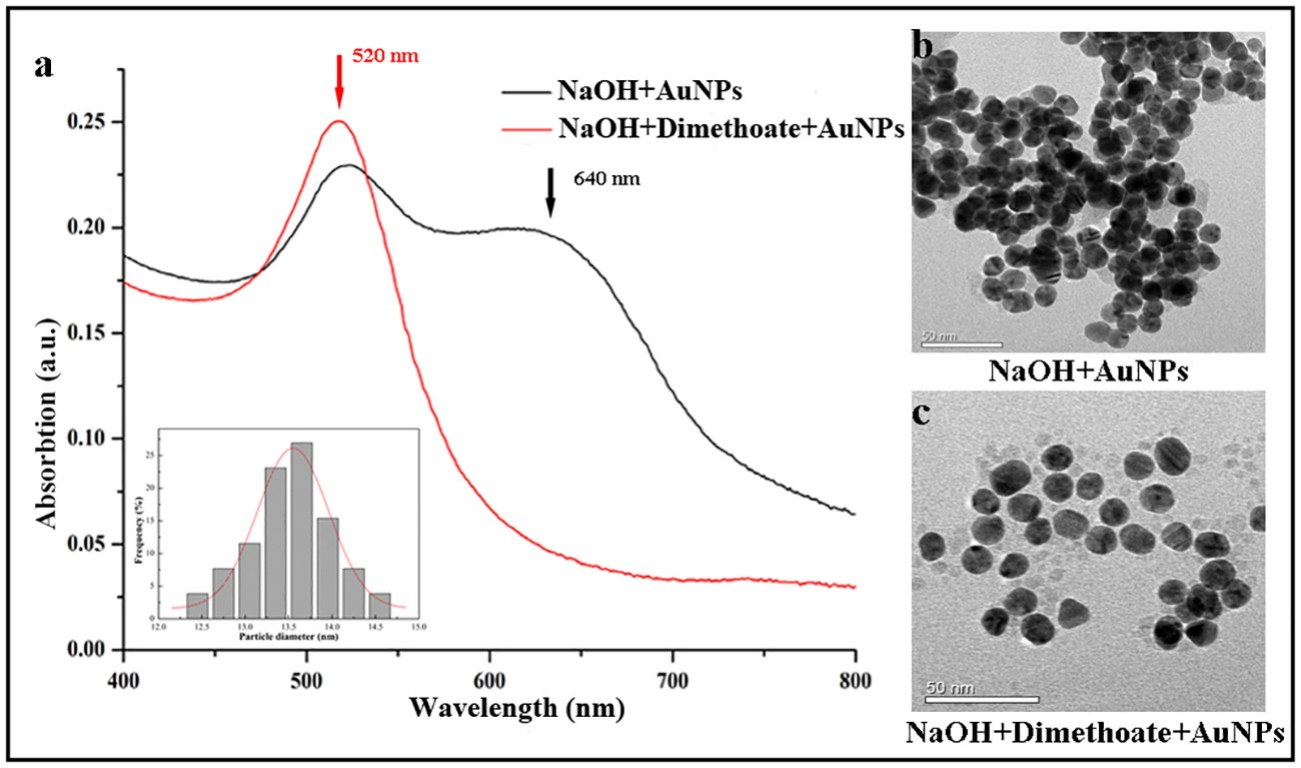
The absorption spectra (a) and TEM images of the NaOH+AuNPs solutions with and without dimethoate (0.1 μM) (b and c).

The dimethoate concentration used for the experiment was 0.1 μM. The morphology of AuNPs was characterized by a TEM. A typical TEM image of AuNPs and absorption spectrum measured by the UV-Vis spectrophotometer are presented in Fig 2. From Fig 2a, an absorption peak centered at 520 nm was obtained from the solution with dimethoate (the red curve). In this case, the AuNPs were still well dispersed in the mixture solution (the inset Fig 2c). This phenomenon resulted from repulsion between the negative charges possessed by the hydrolyzed product of dimethoate, an organophosphorus pesticide and the AuNPs. On the contrary, the measured solution without dimethoate not only have two absorption peaks at 520 nm and 640nm, respectively, but also the color of the AuNPs solution turns into blue due to the aggregated AuNPs (the inset Fig 2b). These experimental results displayed the potential application of the LSPR phenomenon in dimethoate detection.

### Stability of the dual-channel LSPR system

AuNPs with the size of 13.5 nm were selected for this experiment. AuNPs solution with the concentration of 9.5 nM was diluted 5 times. 330 uL of the AuNPs solution was put into the cuvettes for 6 repeated measurement. The results of the absorption maximum at 520 nm were obtained from this dual-channel LSPR system (Table 1). From Table 1, the average error calculated from both channels has a slight difference. It confirmed the two samples can be measured in parallel after calibration curves are established.

**Table 1:** Stability of the dual-channel LSPR system.

	A_max_(520)						Average error
	1	2	3	4	5	6	
Ch1	0.329	0.329	0.326	0.330	0.327	0.330	0.004
Ch2	0.316	0.316	0.313	0.319	0.317	0.316	0.006

### Optimization of experimental conditions

**Fig 3:**
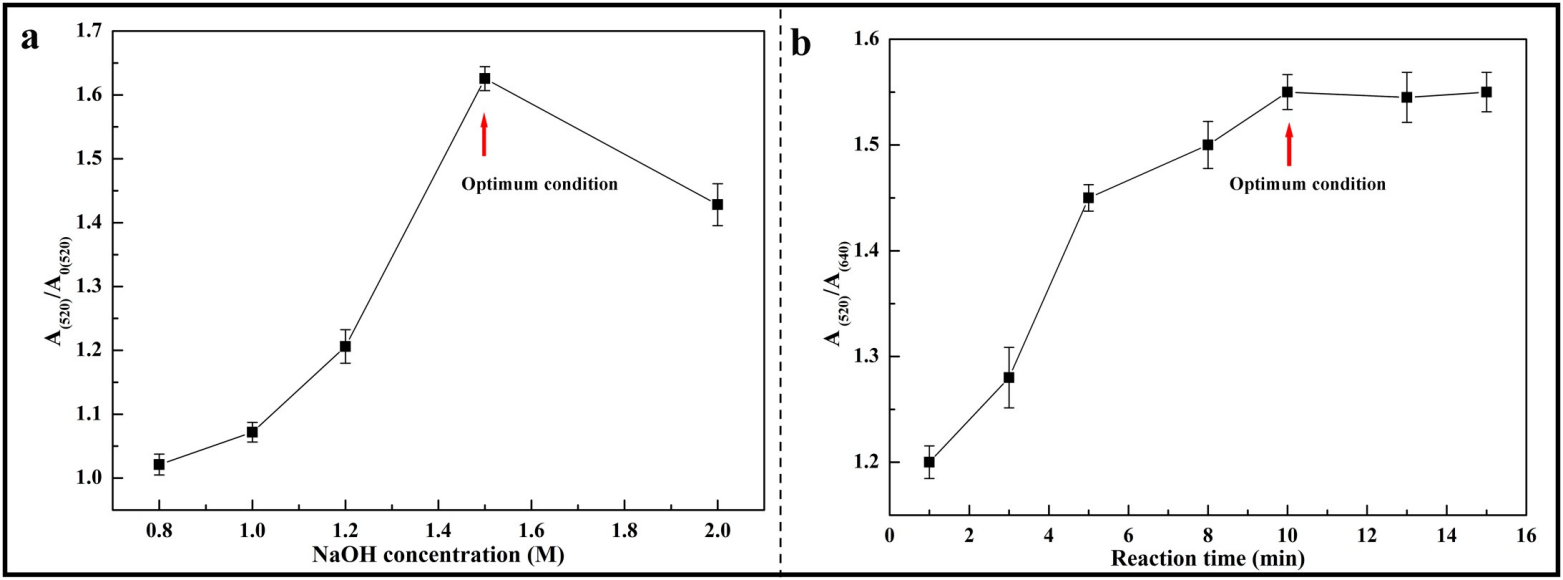
Figure showing the optimization of NaOH concentration (a) and reaction time (b) for dimethoate detection.

Sodium hydroxide (NaOH) concentration plays a significant role in this experiment and directly affects the sensitivity of the results. A high concentration of NaOH can make the AuNPs aggregate quickly. Therefore, the concentrations of NaOH used in this research work were optimized accordingly. Obviously, the reaction time determined the stability of chemical reaction process and the accuracy of the experimental results. For the optimization of NaOH concentration, five different concentrations i.e. 0.8 M, 1.0 M, 1.2 M, 1.5 M and 2.0 M of NaOH were used, respectively. Firstly, 80 μL of dimethoate (0.1 μM) were added into a cuvette with 535 μL Milli-Q water. Then, 150 μL AuNPs was added into this solution mixture. Finally, 35 μL of NaOH with above different concentrations (0.8 M, 1.0 M, 1.2 M, 1.5 M and 2.0 M) was placed into the solutions. A plot of the ratio of the absorbance value of the mixture (NaOH and dimethoate) to that of pure NaOH at wavelength of 520 nm, A(520)/A_0_(520) versus NaOH concentration were done and displayed in Fig 3a respectively. It was observed that the ratio reached its maximum when the concentration of NaOH was 1.5 M. Therefore, 1.5 M of NaOH concentration was chosen as the optimized value. For determination of the optimum reaction time, 35 μL of NaOH (1.5M) and 80 μL dimethoate solution (0.1 μM) were mixed with 535 μL Milli-Q water and 150 μL AuNPs. The reactions were recorded at different time, such as 1 min, 3 min, 5 min, 8 min, 10 min, 13 min and 15 min. A plot of the ratio A(520)/A (640) versus reaction time was also done (Fig 3b). It was found that the absorbance ratio increased with increase in reaction time with a plateau being achieved after a reaction time of 10 min. This reaction time i.e. 10 min, was therefore, confirmed as the optimum reaction time.

### Quantitative detection of dimethoate using the dual-channel LSPR system

**Fig 4:**
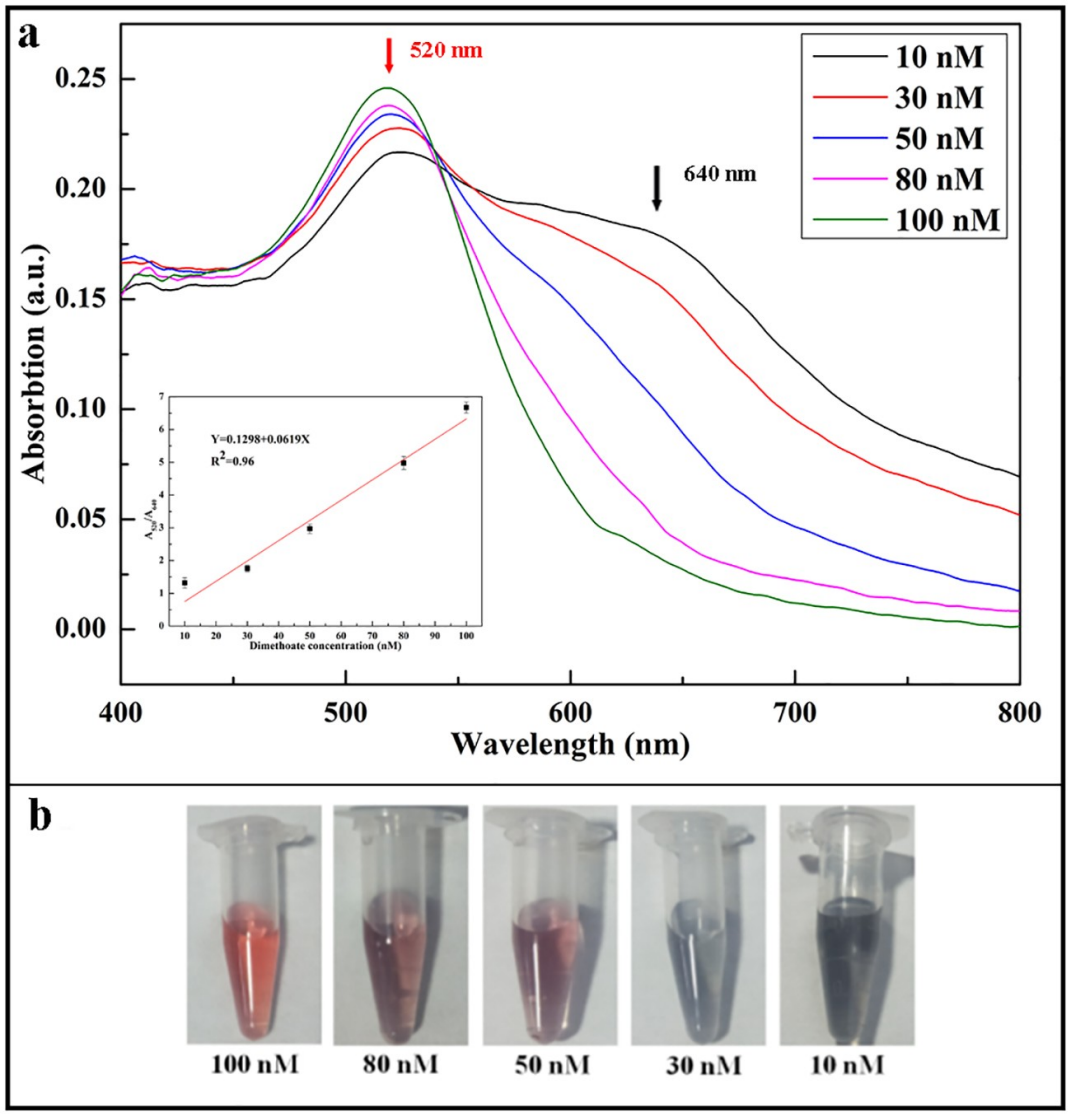
Quantitative detection of dimethoate using the Ch1 in the dual-channel LSPR system. Absorption spectra (a), the inset calibration curve and color of the measured samples in vitals (b) with different concentrations of dimethoate in the range of 10–100 nM.

**Fig 5:**
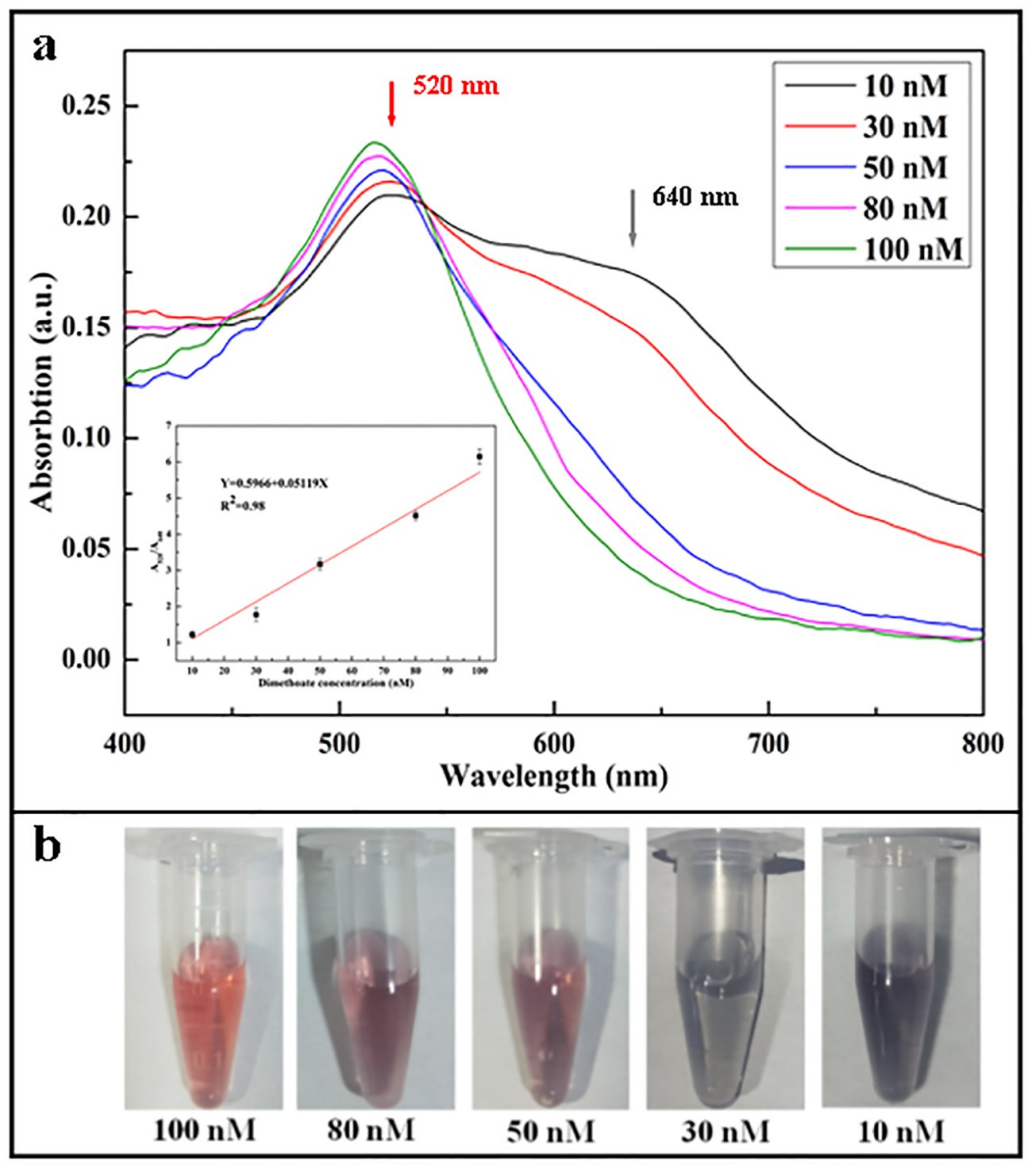
Quantitative detection of dimethoate using the Ch2 in the dual-channel LSPR system. Absorption spectra (a), the inset calibration curve and color of the measured samples in vitals (b) with different concentrations of dimethoate in the range of 10–100 nM.

Under the optimized conditions described above, 80 μL of dimethoate with different concentrations was added into 535 μL Milli-Q water, 150 μL AuNPs was added into the mixture (solution), then 35 μL of NaOH was added and mixed thoroughly. After obtaining a homogenous mixture, it was left to react at room temperature for 10 min. Using the dual-channel fiber optic LSPR system and the SpectroSuite software, the absorption spectra were recorded with an exposure time of 3s per scan and an average of three scans. The spectral window of 400 nm to 800 nm and the boxcar smoothness window of 5 were used. A reference spectrum (chosen as the background) was measured after inserting a cuvette containing 330 μL of pure Milli-Q water into the holder of Ch1. The other cuvette with 330 μL of mixture (sample containing dimethoate) was put into the holder of Ch2 to detect the absorption separately. Each measurement were repeated three times and the average absorption spectrum of the solution plotted (Figs 4a and 5a). These results indicated that by increasing the concentration of dimethoate pesticide, the absorbance at 520 nm gradually increased while it gradually decreased at 640 nm. The color of the mixture (i.e. solution) changed from blue to red (Figs 4b and 5b). The results also shows that there were linear correlations between dimethoate concentrations and the absorbance ratio A(520)/A(640) in the concentration range of 10 nM to 100 nM. A linear correlation coefficient of 0.97 and a limit of detection (LOD) of 5.5 nM was obtained from the measurement results of Ch1, Ch2, which was determined by the formula 3σ/slope, where σ is the standard deviation of blank, i.e. the absorbance of pure AuNPs and slope can be obtained from the linear calibration curve [31] (the inset Fig 4a and inset Fig 5a). From these experiment results, there are certain systematic errors existed in both channels. These systematic errors were minimized by the calibration curves established from both channels to meet the detection requirements.

### Specificity of the dual-channel LSPR system

**Fig 6:**
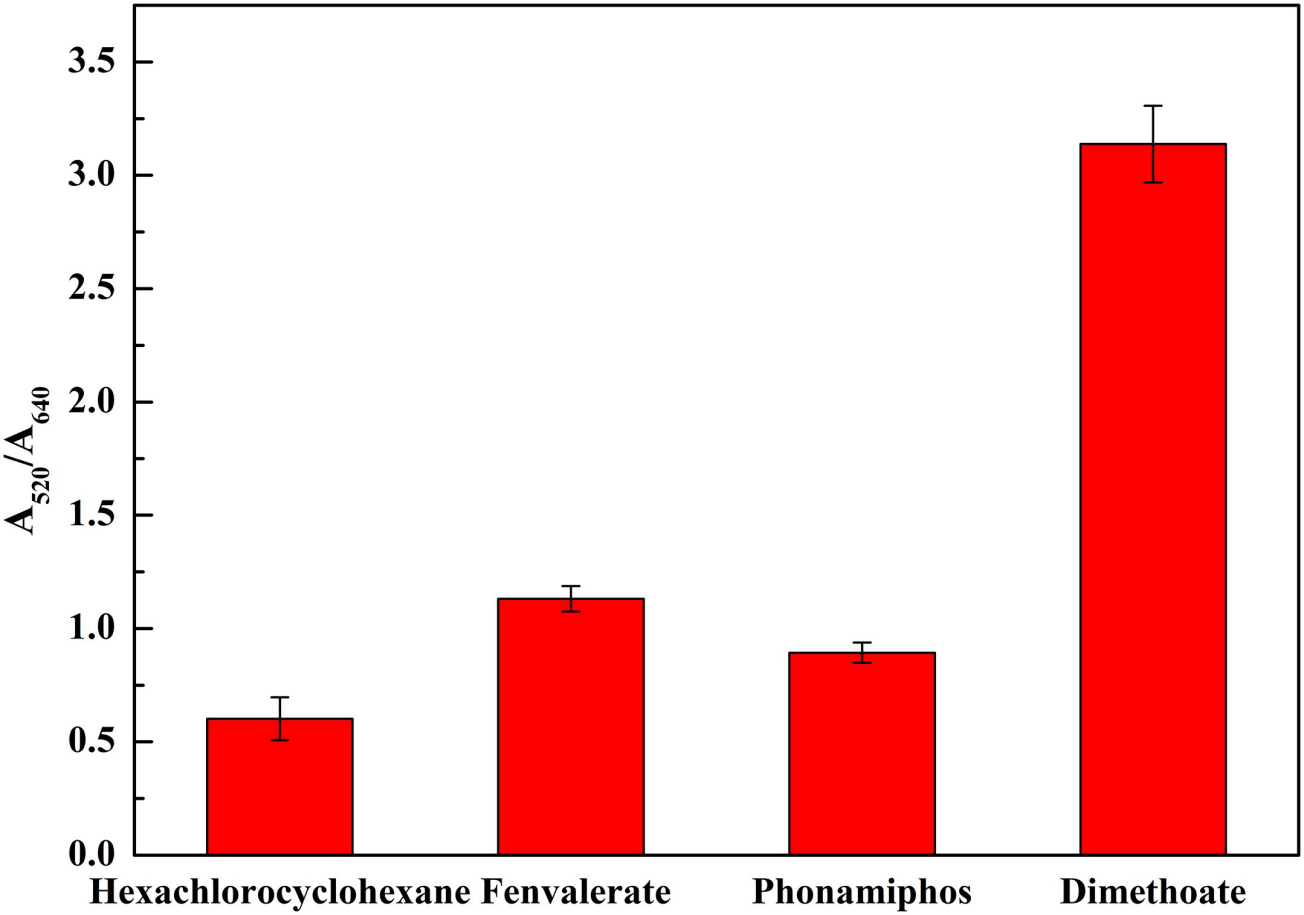
Selectivity of the dimethoate (0.05 μM) detection compared to several other non-target pesticides (0.5 μM).

The selectivity of the proposed method for dimethoate detection was also evaluated using several other non-target pesticides that included Hexachlorocyclohexane, Fenvalerate and Phonamiphos. A concentration of 0.5 μM of these non-target pesticides (e.g. Hexachlorocyclohexane, Fenvalerate, Phonamiphos) and 0.05 μM of dimethoate were respectively detected by this method. The results showed that the non-target pesticides could not be hydrolyzed by NaOH and the color of mixed solution turned blue quickly (Fig 6). However, the color of the AuNPs solution containing dimethoate was red rather than blue and the absorbance ratio of 3.138 were obtained, which was the highest among four pesticides. Clearly, these results showed that the method was highly sensitive for the detection of dimethoate pesticides. The anti-aggregation method based on AuNPs were highly selective for detecting organophosphorus pesticides, such as dimethoate, because most organophosphorus pesticides contain phosphate ions which could be hydrolyzed under strong alkali conditions. The molecular weight and structure of each organophosphorus pesticide were totally different, resulting in molar cross-section extinction coefficient differently. The LSPR response curves were specific to the structures of the organophosphorus pesticides.

### Detection of dimethoate in apple samples

To study the reliability and efficacy of this method in environmental samples, the apple samples were detected using both Ch1 and Ch2. Preparation of the apple samples involved the extraction of the juice, centrifuging for 10 min at 12000 r/min and finally filtered using a 0.22 μm filter. Dimethoate with different concentrations of 20 nM, 40 nM, and 90 nM were mixed separately with the apple juice. Consistent with the above-mentioned method, detection of dimethoate in apple juice was done using Ch1 in this LSPR system. The experimental recovery rates are given in Table 2. The actual detected concentrations in all three samples of Ch1 were calculated as 17.18 nM, 37.64 nM and 94.45 nM, respectively. The results showed that the detection recovery rate of dimethoate was 85.90% to 105.44% in the concentration ranged of 10 nM to 100 nM. The experimental results showed that Ch1 is feasible to be used to detect dimethoate in environmental samples. In Ch2, the actual detected concentrations were calculated as 18.58 nM, 42.15 nM and 96.63 nM. The detection recovery rates for this channel were between 92.90% to 107.37%, with a similar dimethoate concentration as in Ch1. The results showed that both channels were equally good in detecting dimethoate in apple samples.

**Table 2:** Experimental results of dimethoate detection recovery rates in apples.

	Sample	Dimethoate concentration (nM)		Detection recovery rate (%)
		The known concentration	Detection results	
Ch1	1	20	17.18	85.90
	2	40	37.64	94.10
	3	90	94.45	105.44
Ch2	1	20	18.58	92.90
	2	40	42.15	105.38
	3	90	96.63	107.37

### Comparison of this method with the existing methods

A comparative analysis between different other techniques and their efficiencies were displayed in Table 3. Absorbptionmetric method with AChE on gold nanorods is labor-intensive and low detection limit. Colorimetric with AgNPs method were detect rapidly and wide detection ranges. Electrochemical workstation with AChE method were highly reproducibility and very expensive. SERS method is identification effectively with high detection limit. Fluorescent hydrogel method is accuracy, stable and complex operation in the laboratory. It can be seen that the dual channel LSPR system demonstrated here has more advantages especially in terms of LOD, portability, dual detection and cost. The system has a great potential for being developed into a low cost and portable tool for the detection of dimethoate in the future. Both Ch1 and Ch2 in this LSPR system displayed good linear relationship between dimethoate concentration within the range 10–100 nM and the absorbance ratio A(520)/A(640) giving correlation coefficients (R^2^) of 0.97. Besides, they displayed detection limits of 5.5 nM.

**Table 3:** Comparison of the existing methods with our dual channel LSPR method.

Methods	Detection range	LOD	Properties	References
Absorbptionmetric method with AChE on gold nanorods	5 nM-1μM	3.9 nM	• Labor-intensive	Lang et al., Talanta, (2016) [2]
			•Low detection limit	
Colorimetric with AgNPs	0.5–88 mg/kg (≈3.05–636.8 μM)	0.5 mg/kg (≈3.05 μM)	•Detect rapidly	Li et al., (2015) [32]
			•Wide detection ranges	
Electrochemical workstation with AChE	1.75–10 μM	10 nM	•High reproducibility	Guan et al., Advanced Materials Research, (2011) [33]
			•Expensive	
SERS using portable instrumentations	0.5–10 μM	0.5 μM	•Identification effectively	Tognaccini et al., Molecules (Basel, Switzerland), (2019) [34]
			•High detection limit	
Fluorescent hydrogel	0.001–5 mg/L (≈4.36–21.8 pM)	1 μg/L (≈4.36 pM)	•Accuracy and stability	Deshuai et al., Biosensors and Bioelectronics, (2019) [35]
			•Complex operation	
Dual-channel Absorptiometric System	10–100 nM	5 nM	•Portable and low cost	This work
			•Multichannel detection	
			•Low detection limit	

## Conclusion

This study demonstrates high sensitivity, dual-channel LSPR system via optical fibers to detect dimethoate. The surface of the synthesized AuNPs carries negative charge uniformly under various concentrations and reaction times in the AuNPs solution. Using LSPR spectrum analysis, chemically NaOH and reaction time were optimized to detect dimethoate based on anti-aggregation. Subsequently, AuNPs were uniformly disperse into the solution via adding NaOH for 10 min to produce the final plasmon active background. A portable transmission-mode non-functionalized LSPR system was then produced by optical fibers connected to the cuvette cell with SMA connectors. The detection performance of the dual-channel LSPR system developed in this study was evaluated using apply juice as a real sample. Under these optimized conditions, the relationship between concentration of dimethoate and absorbance ratio (A(520) /A(640)) was obtained. The experimental results from Ch1 and Ch2 showed that there was a good linear relationship between concentration of dimethoate and the absorbance ratio (A(520) /A (640)) in the range of 10 nM to 100 nM dimethoate concentration with the linear correlation coefficient of 0.97 and the limit of detection (LOD) of 5.5 nM. The detection specificity and the recovery rate of this LSPR system were evaluated for various dimethoate concentrations in apple juice. It was found that detection recovery rates of this dimethoate LSPR system was obtained between 85.90% to 107.37%. Although there were slight differences in recovery rate between both channels, the calibration curves can be sued to reduce the error from structure of channel and obtain the reliable experimental results. The developed dual-channel LSPR system via optical fiber allows to fast detect the dimethoate and greatly improve the detection range of dimethoate. It could establish a novel approach to develop a high-throughput analysis of the insecticides using a wavelength division multiplexing switch (WDMS) in various fields such as medical diagnosis, environmental monitoring, and food safety.

## Supporting information

S1 Data (ZIP)
